# Evaluation of the Impact of a Mobile App (LoAD Calc) on the Calculation of Maximum Safe Doses of Local Anesthetics: Randomized Controlled Trial

**DOI:** 10.2196/89236

**Published:** 2026-07-30

**Authors:** Alexandra Stefani, Pietro Elias Fubini, Tal Sarah Beckmann, Caroline Flora Samer, Georges Louis Savoldelli, Mélanie Suppan

**Affiliations:** 1 Division of Anaesthesiology, Department of Acute Care Medicine, Geneva University Hospitals, Rue Gabrielle-Perret-Gentil 4, Geneva, 1205, Switzerland, 41 3723311; 2 Division of Clinical Pharmacology and Toxicology, Department of Acute Care Medicine, Geneva University Hospitals, Geneva, Switzerland; 3 Department of Anaesthesiology, Pharmacology, Intensive Care and Emergency Medicine, Faculty of Medicine, University of Geneva, Geneva, Switzerland

**Keywords:** local anesthetics, dose calculation, toxicity, mobile apps, safety

## Abstract

**Background:**

Local anesthetics (LAs) are widely used in clinical practice, but their administration carries the risk of LA systemic toxicity, a potentially fatal complication. Calculating maximum safe doses is complex and error-prone when considering multiple patient-specific factors. Most existing tools and mobile apps have not been scientifically validated and may propose unsafe doses. The Local Anesthetics Dose Calculator (LoAD Calc) mobile app was developed at the Geneva University Hospitals to calculate maximum safe doses by integrating comprehensive patient parameters.

**Objective:**

The primary objective was to evaluate the overdose rate using the LoAD Calc app compared with traditional calculation methods. Secondary objectives were calculation time, user confidence, app usability, and interpractitioner variability.

**Methods:**

This single-center unblinded randomized controlled trial recruited 50 anesthesiologists to use either LoAD Calc or their usual calculation methods. Assessment was conducted in-person using a web-based platform. Participants completed 10 clinical vignettes featuring various patient characteristics, comorbidities, and medication interactions requiring maximum safe dose calculations for lidocaine, levobupivacaine, and ropivacaine. The primary outcome was the overdose rate, defined as exceeding the maximum safe doses calculated using a comprehensive set of rules incorporating all patient-specific factors. Secondary outcomes included overdose rates using 2 simplified weight-based methods, underdose rates, calculation time, and self-reported measures of confidence and app usability. Overdose rates were compared using chi-square tests with 95% CIs; calculation time and confidence were assessed using Student *t* test.

**Results:**

All 50 participants completed the study. Using the full set of calculation rules, the overdose rate was significantly lower when LoAD Calc was used (30/250, 12.0% vs 198/250, 79.2%; relative risk [RR] 0.15, 95% CI 0.11‐0.21; *P*<.001). This was consistent across all 3 LAs: ropivacaine (5/100, 5% vs 64/100, 64%; *P*<.001), levobupivacaine (19/100, 19% vs 90/100, 90%; *P*<.001), and lidocaine (6/50, 12% vs 44/50, 88%; *P*<.001). When considering simplified weight-based methods, overdose rates remained significantly lower when LoAD Calc was used (actual weight: 8/250, 3.2% vs 95/250, 38.0%; RR 0.08, 95% CI 0.04‐0.17; *P*<.001; ideal body weight: 10/250, 4.0% vs 151/250, 60.4%; RR 0.07, 95% CI 0.04‐0.12; *P*<.001). Underdose rates were similar (25/250, 10.0% vs 27/250, 10.8%; *P*=.77). Calculation time was also similar (mean 111, SD 56 s vs mean 115, SD 89 s; *P*=.53). LoAD Calc users reported higher confidence (mean 8.5, SD 0.9 vs mean 6.3, SD 1.7; *P*<.001), and the app achieved a median System Usability Scale score of 98 (IQR 95-100).

**Conclusions:**

LA overdoses were significantly less likely when the LoAD Calc app was used in simulated clinical vignettes, without increasing calculation time or underdose rates. Given its high usability score and the improvement in practitioner confidence, clinical implementation could be considered and may contribute to improving patient safety and dose calculation standardization.

## Introduction

Local anesthetics (LAs) are widely used by clinicians across various settings, including operating rooms and office-based procedures [1,2]. In anesthesia practice, local and regional anesthesia have become integral components of perioperative management for elective surgical procedures [3]. While generally safe and effective, LA overdose can expose patients to serious complications, particularly local anesthetic systemic toxicity (LAST), which manifests as neurological and cardiac toxicity and can be potentially fatal [4,5].

To prevent LAST, the American Society of Regional Anesthesia and Pain Medicine (ASRA) recommends several safety measures, including ultrasound guidance, incremental and fractional injection with intermittent aspiration, the use of intravascular markers, and the administration of the lowest effective LA dose [6].

In clinical practice, dose calculations are typically performed according to empirical guidelines, with many clinicians relying on mental calculations or basic calculators. However, the mental calculation of maximum safe LA doses is challenging and error-prone, particularly when multiple variables must be considered simultaneously, including patient weight, age, comorbidities, LA type and concentration, injection site vascularity, and potential drug interactions [7,8]. When LA mixtures are used, complexity grows substantially, thereby significantly increasing the risk of calculation errors. To address this challenge, tools such as nomograms and mobile apps have been developed to facilitate dose calculations [9]. However, most have not been scientifically validated for clinical implementation, and preliminary assessments suggest that they allow the calculation of doses that could exceed safe limits and potentially lead to LAST [10].

A mobile health (mHealth) app, Local Anesthetics Dose Calculator (LoAD Calc), was developed at the Geneva University Hospital to assist anesthesiologists in calculating safe maximum LA doses [10]. This app integrates various patient parameters and enables calculations for LA mixtures. The hypothesis was that automating LA dose calculation would reduce overdose rates without increasing the risk of underdosing or calculation time. The primary objective of this study was to evaluate whether the LoAD Calc app reduces overdose rates compared to traditional calculation methods. Secondary objectives included assessing the impact on calculation time, user confidence, app usability, and whether the app standardizes LA dosing in clinical practice.

## Methods

### Study Design and Participants

This single-center, parallel-group randomized controlled trial was conducted at the Geneva University Hospitals. The study protocol was previously published [11]. This paper was designed according to the CONSORT-EHEALTH (Consolidated Standards of Reporting Trials of Electronic and Mobile Health Applications and Online TeleHealth) checklist (Checklist 1) [12]. Relevant elements from the CHERRIES (Checklist for Reporting Results of Internet E-Surveys) were included due to the use of online questionnaires [13]. The trial was conducted in an unblinded design, as it was not possible to conceal the intervention type from participants.

All resident physicians and registrars in the anesthesiology department were eligible for participation. All participants were employed at the Geneva University Hospitals, a tertiary academic center, and were therefore subject to the same institutional workload and mandatory continuing medical education requirements. Regional anesthesia is routinely performed as part of standard perioperative care at this institution. The sole exclusion criterion was current or prior use of the LoAD Calc app, determined through screening questions.

### Ethical Considerations

The regional ethics committee (Commission Cantonale d’Éthique de la Recherche [CCER], Geneva, Switzerland) issued a declaration of no objection (CCER 2022‐01577), as the study did not fall within the scope of Swiss federal law on human research [14]. This exemption was appropriate as the study involved only health care professionals in a simulated setting, with no patient data collected. All participants received a written information sheet and provided explicit consent before participating. Consent was obtained through an online platform, where participants clicked an “I agree to participate” button before proceeding. They were informed that participation was entirely voluntary and that they could withdraw at any time without consequence. Unique anonymous identifiers were used throughout; no personal identifying information was linked to study responses. The investigator (MS) who analyzed the data had no means of linking the anonymous identifiers to individual participants, and the final dataset used for analysis was fully anonymized. Participants received no financial compensation or other material incentives. The only benefit offered was the advancement of scientific knowledge.

### LoAD Calc App Description

LoAD Calc is a multiplatform mobile app (iOS and Android) developed at the Geneva University Hospitals, as previously described in detail [10]. Briefly, the user selects the LAs and their concentrations, enters the patient’s weight and height, and selects sex and the presence of relevant dose-modifying factors, including age over 70 years, comorbidities (renal insufficiency defined as glomerular filtration rate <50 mL/min, hepatic insufficiency defined as prothrombin time <50%, severe heart failure defined as left ventricular ejection fraction ≤30%, and pregnancy), and concomitant medications known to decrease LA metabolism (major CYP1A2 and CYP3A inhibitors). If one dose-modifying factor is present, the maximum dose is reduced by 20%; if 2 or more are present, it is reduced by 30%. The results are displayed as maximum safe volumes in milliliters, which is the clinically relevant unit for anesthesiologists. The app works offline and was developed using the Dart programming language with the Flutter open-source development kit (Google LLC). Screenshots of the app interface are provided in Multimedia Appendix 1. Version 1.2.1 of LoAD Calc was evaluated in this trial, and no updates or changes were made to the app’s functionality or content during the study period. The complete calculation rules and algorithms are detailed in this paper and the referenced development paper [10]. The source code is available upon request from the corresponding author.

### Clinical Vignettes

Ten clinical vignettes (Multimedia Appendix 2) were developed by one of the authors (PEF), featuring realistic scenarios requiring LA dose calculations for 3 commonly used agents: lidocaine, levobupivacaine, and ropivacaine. The vignettes included various patient characteristics, comorbidities, and medication interactions requiring dose adjustments. These vignettes have already been described in a previous paper [15]. For each vignette, 3 authors (PEF, TSB, and MS) were required to determine the maximum dose of LA the simulated patient should receive according to the rules used to develop the LoAD Calc app, without using the app. Any disagreement prompted a review of the vignette. Final vignette approval was only possible if a consensus was reached.

### Sample Size and Randomization

The sample size calculation was performed using Stata (version 17.0; StataCorp LLC). A minimum clinically meaningful difference of 10% in overdose rates was assumed based on clinical judgment, with an SD of 10% and a power of 90%, yielding a required sample size of 46 participants (23 per group). This was rounded to 50 to account for potential attrition.

Participants were randomized into one of 2 groups. The control group used their usual methods for calculating maximum safe LA doses, while the LoAD Calc group used the LoAD Calc mobile app preinstalled on a standardized dedicated smartphone. Randomization was stratified based on participants’ positions (resident vs registrar) using Stata’s balanced randomization mechanism by MS. Unique identifiers consisting of 12 random alphanumeric characters were generated and prerandomized to the respective groups. These identifiers served as login credentials for the web-based platform (described later) and automatically assigned participants to their respective groups (resident vs registrar and LoAD Calc vs traditional method).

### Recruitment

Participants were recruited one at a time from the anesthesiology department of the Geneva University Hospitals between February 2024 and February 2025 and were temporarily relieved from their clinical duties. Participants were enrolled by MS, TSB, AS, and PEF. Potential participants first received an information sheet summarizing the study details and data protection measures. The information sheet did not identify which calculation method was the intervention of interest, maintaining a neutral presentation to minimize participant bias regarding the comparison. If they consented to participate, they were accompanied by an investigator to a dedicated room equipped with a computer. Participants were asked to put their personal phones in airplane mode and to give their professional phones to a colleague to avoid interruptions during the approximately 1-hour session. They were then asked to randomly choose a sealed, opaque envelope containing the credentials necessary to log in.

### Online Platform

A dedicated web-based platform was developed by one of the authors (MS) using the Joomla! 4.3 content management system (Open Source Matters). The platform was hosted on a Swiss server (Kreativmedia GmbH) and secured using RSFirewall 3 (RSJoomla) and AdminTools 7 (Akeeba Ltd). Prior to implementation, the platform design and questionnaire interface were reviewed and tested by all authors to ensure usability and technical functionality. The study home page provided general information about the research and enabled participants to login. An initial questionnaire, managed using Shondalai Community Surveys 6 (Bulasikku Technologies Pvt Ltd), facilitated participant consent collection, exclusion criterion verification, and demographic data gathering. Then, an introductory screen displayed information regarding the clinical vignettes participants were about to encounter and specified the calculation method they were to use (LoAD Calc app for the experimental group vs the participant’s preferred method for the control group). At this stage, those allocated to the LoAD Calc group were given a smartphone preinstalled with the LoAD Calc app. Participants received no prior training or instruction on the app and used it for the first time during the study session. The clinical vignettes were presented one by one in randomized order using Shondalai Community Quiz 7. A timer automatically started once each vignette page was loaded and stopped upon validation of the entered dose. After completing the vignettes, participants allocated to the LoAD Calc group completed the validated French version of the System Usability Scale (SUS) [16,17]. Participants allocated to the control group were asked which methods they used to calculate LA doses. Finally, both groups answered a question based on a 10-point Likert scale to assess their confidence in the method they used to carry out the maximum safe LA dose calculations, ranging from 1 (absolutely not confident) to 10 (perfectly confident). All participants were invited to provide open-ended comments or feedback about their experience with the calculation method or app if they wished to do so.

All data were stored in an encrypted MySQL-compatible database (MariaDB 10, MariaDB Foundation).

### Overdose and Underdose Definitions

When using LAs, anesthesiologists decide to administer a specific volume (mL) rather than a particular quantity (mg). Thus, although toxicity is related to the quantity of LA used, it is clinically more relevant to determine the maximum volume (mL) of LA that should be used for a particular case.

To determine the maximum safe dose of LA for each vignette, quantities (mg) were computed using 3 different calculation methods before being converted to milliliters according to the LA’s concentration. The comprehensive method utilized the full set of calculation rules incorporated in the LoAD Calc app as previously described [10], including patient-specific factors such as age, comorbidities, and drug interactions. Two simplified methods were also employed: one based on actual weight (AW) and the other on ideal body weight (IBW) using the usual formula:


Maximum dose (mg)=Weight (AW∨IBW)(kg)×Dose limit for chosen LA(mg/kg)

This multimethod approach reflects the complexity of LA dosing in clinical practice, where various calculation methods coexist. While the comprehensive, most conservative method served as the primary evaluation criterion due to its rigorous safety assessment, the simplified methods were used to reflect common clinical practices, where weight-based calculations are more commonly employed without taking other factors into account [18].

If the volume obtained by converting the maximum safe quantities of LA into milliliters was an integer, it was considered the maximum safe volume without any further change. If the result was a decimal value, it was first rounded down to the nearest integer to be consistent with the rules used to develop the LoAD Calc app. However, to avoid being too conservative, a value of 1 mL was then added, and the final value (either in milliliters or its equivalent in milligrams) was considered the maximum safe dose for the purpose of this study. Any dose higher than this value was considered an overdose. All maximum safe doses are reported in Table 1.

An underdose was defined as the maximum acceptable volume minus 20% (or its corresponding LA quantity in milligrams), rounded down to the nearest integer. This was an empirical choice since anesthetic underdose can only be determined clinically.

**Table 1.** Patient characteristics and maximum safe local anesthetic doses according to actual weight (AW), ideal body weight (IBW), and the full set of calculation rules.

Vignette (unit)	Weight (kg)	Height (cm)	Local anesthetic assessed	Mixture	Relevant characteristics	Maximum safe dose		
						Full rules	AW	IBW
1 (mg)	55.0	174	Ropivacaine	No	None	165	165	210
2 (mL)	51.5	159	Levobupivacaine	Yes	>70 years	10	14	14
3 (mg)	60.0	170	Lidocaine	Yes	Pregnancy	50	80	90
4 (mL)	70.0	170	Levobupivacaine	No	>70 years, renal failure	19	28	27
5 (mg)	120.0	180	Levobupivacaine	No	CYP1A2 inhibitor	115	240	145
6 (mL)	80.0	175	Ropivacaine	Yes	Liver cirrhosis	19	40	29
7 (mg)	60.0	175	Levobupivacaine	No	>70 years, heart failure	86.25	120	142.5
8 (mL)	75.0	170	Lidocaine	Yes	None	8	12	8
9 (mg)	90.0	160	Ropivacaine	No	>70 years	127.5	270	157.5
10 (mL)	80.0	157	Ropivacaine	No	None	30	48	30

### Outcome Measures

The primary outcome was the overall overdose rate computed using the full calculation rules. The secondary outcomes were the overall overdose rate according to AW and IBW, the overdose rate according to each LA studied, the overall underdose rate, and the variability of results between participants.

The time taken to complete these calculations, the app’s usability (according to the SUS), and the physicians’ confidence (on a score ranging from 0 to 10) in the method they used to determine the maximum safe LA doses were also analyzed.

### Statistical Analysis

Data were extracted from the encrypted MySQL-compatible database (MariaDB) into CSV files and imported into Stata 19 BE (StataCorp LLC) for data curation and statistical analysis. The primary statistical analysis was conducted by MS, who was blinded to group assignment.

Descriptive statistics were used to detail participants’ characteristics. Normality was checked first graphically and then, in case of doubt, using the Shapiro-Wilk test. Values were reported accordingly.

The chi-square test was used to compare overdose and underdose rates; risk ratios with 95% CIs were also reported. The time required to complete the vignettes and participant confidence in the methods they used to determine LA doses were assessed using Student *t* test. Box plots were used to graphically display variability.

A post hoc sensitivity analysis was performed to assess the potential influence of outliers on the primary outcome. Participants with at least 1 dose value exceeding 3 SDs above their group’s mean for that vignette were excluded.

Missing values were reported as such. No imputation method was used. Double-sided *P* values less than .05 were considered significant.

## Results

**Figure 1.**
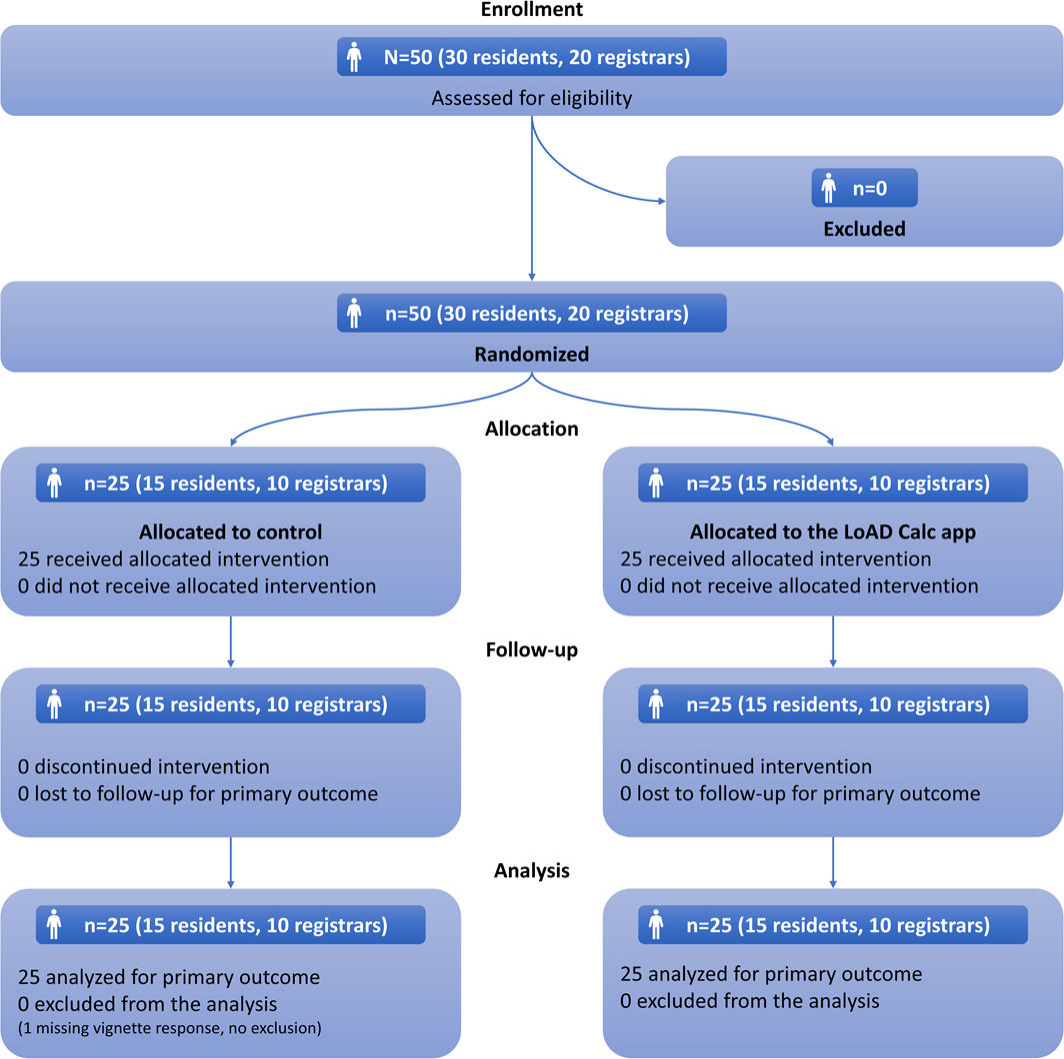
CONSORT (Consolidated Standards of Reporting Trials) flow diagram of participant enrollment, randomization, allocation, follow-up, and analysis. LoAD Calc: Local Anesthetics Dose Calculator.

Fifty anesthesiologists (30 residents and 20 registrars) consented to participate. The flow of participants through the trial is presented in Figure 1. Only 14% (7/50) had already heard about the LoAD Calc app. None of them had ever installed or used the app before participating in the study. Thus, all 50 anesthesiologists were included. Their characteristics are detailed in Table 2.

**Table 2.** Participant characteristics.

Characteristics	Overall (N=50)	Control group (n=25)	LoAD Calc^a^ group (n=25)
Residents, n (%)	30 (60)	15 (60)	15 (60)
Gender (woman), n (%)	23 (46)	10 (40)	13 (52)
Age (y), median (IQR)	34 (32-37)	33 (32-35)	35 (33-38)
Years since diploma, median (IQR)	8 (6-10)	7 (6-10)	9 (6-10)
Years of anesthesiologic practice, median (IQR)	5 (2-7)	5 (3-6)	5 (2-8)

a LoAD Calc: Local Anesthetics Dose Calculator.

One participant, who belonged to the control group, did not answer one of the 10 clinical vignettes. All other participants answered all questions. The proportion of missing data was thus 0.2% (1/500 total responses).

When considering the full calculation rules, the overdose rate was significantly higher in the control group than in the LoAD Calc group (198/250, 79.2% vs 30/250, 12.0%; relative risk [RR] 0.15, 95% CI 0.11‐0.21; *P*<.001). This difference was consistent for all 3 LAs (Table 3).

A post hoc sensitivity analysis, excluding participants with at least 1 dose value exceeding 3 SDs above their group’s mean for that vignette (n=4 in the control group, n=6 in the LoAD Calc group), yielded consistent results for the primary outcome (control: 165/210, 78.6% vs LoAD Calc: 15/190, 7.9%; RR 0.10, 95% CI 0.06‐0.16; *P*<.001).

**Table 3.** Overdose rates according to the full set of calculation rules.

Local anesthetic	Control, n (%)	LoAD Calc^a^, n (%)	Relative risk (95% CI)	*P* value
Ropivacaine (n=100)	64 (64)	5 (5)	0.08 (0.03‐0.19)	<.001
Levobupivacaine (n=100)	90 (90)	19 (19)	0.21 (0.14‐0.32)	<.001
Lidocaine (n=50)	44 (88)	6 (12)	0.14 (0.06‐0.29)	<.001
Overall (N=250)	198 (79.2)	30 (12)	0.15 (0.11‐0.21)	<.001

a LoAD Calc: Local Anesthetics Dose Calculator.

The underdose rate was similar in both groups (27/250, 10.8% in the control group vs 25/250, 10.0% in the LoAD Calc group; RR 0.93, 95% CI 0.55‐1.55; *P*=.77).

When AW was the only parameter taken into account, the overdose rate was 38.0% (95/250) in the control group vs 3.2% (8/250) in the LoAD Calc group (RR 0.08, 95% CI 0.04‐0.17; *P*<.001). A similar difference was observed when considering IBW, with an overdose rate of 60.4% (151/250) in the control group vs 4.0% (10/250) in the LoAD Calc group (RR 0.07, 95% CI 0.04‐0.12; *P*<.001).

There was no difference in the mean time required to complete vignettes (mean 115, SD 89 s in the control group vs mean 111, SD 56 s in the LoAD Calc group; *P*=.53).

Participants felt significantly more confident when using the LoAD Calc app than when using other methods (mean 8.5, SD 0.9 vs mean 6.3, SD 1.7; *P*<.001).

The median SUS score (rated by the 25 participants who used the LoAD Calc app) was 98 (IQR 95-100).

**Figure 2.**
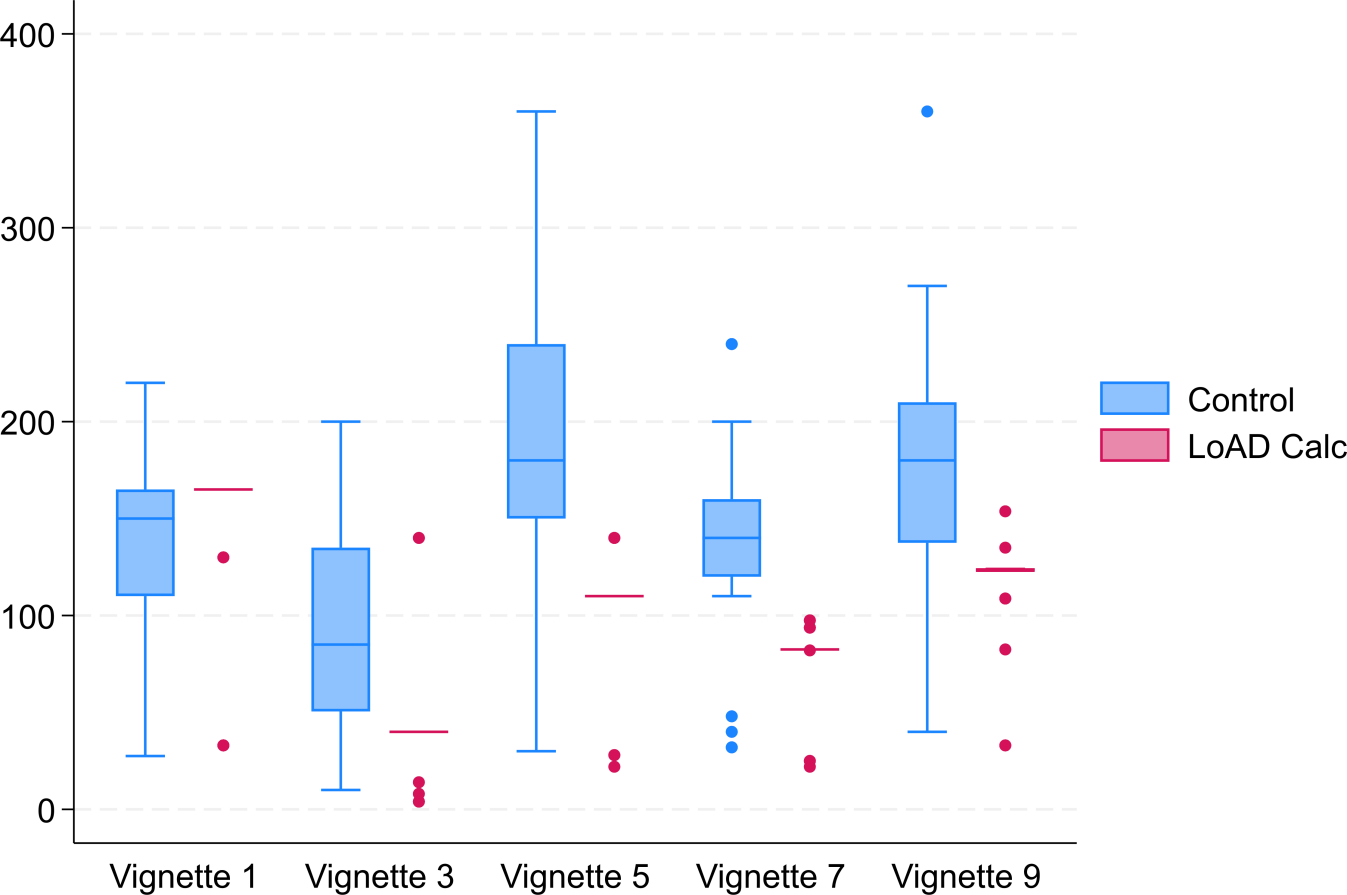
Box plot of the quantities (in mg) recorded by study participants. LoAD Calc: Local Anesthetics Dose Calculator.

**Figure 3.**
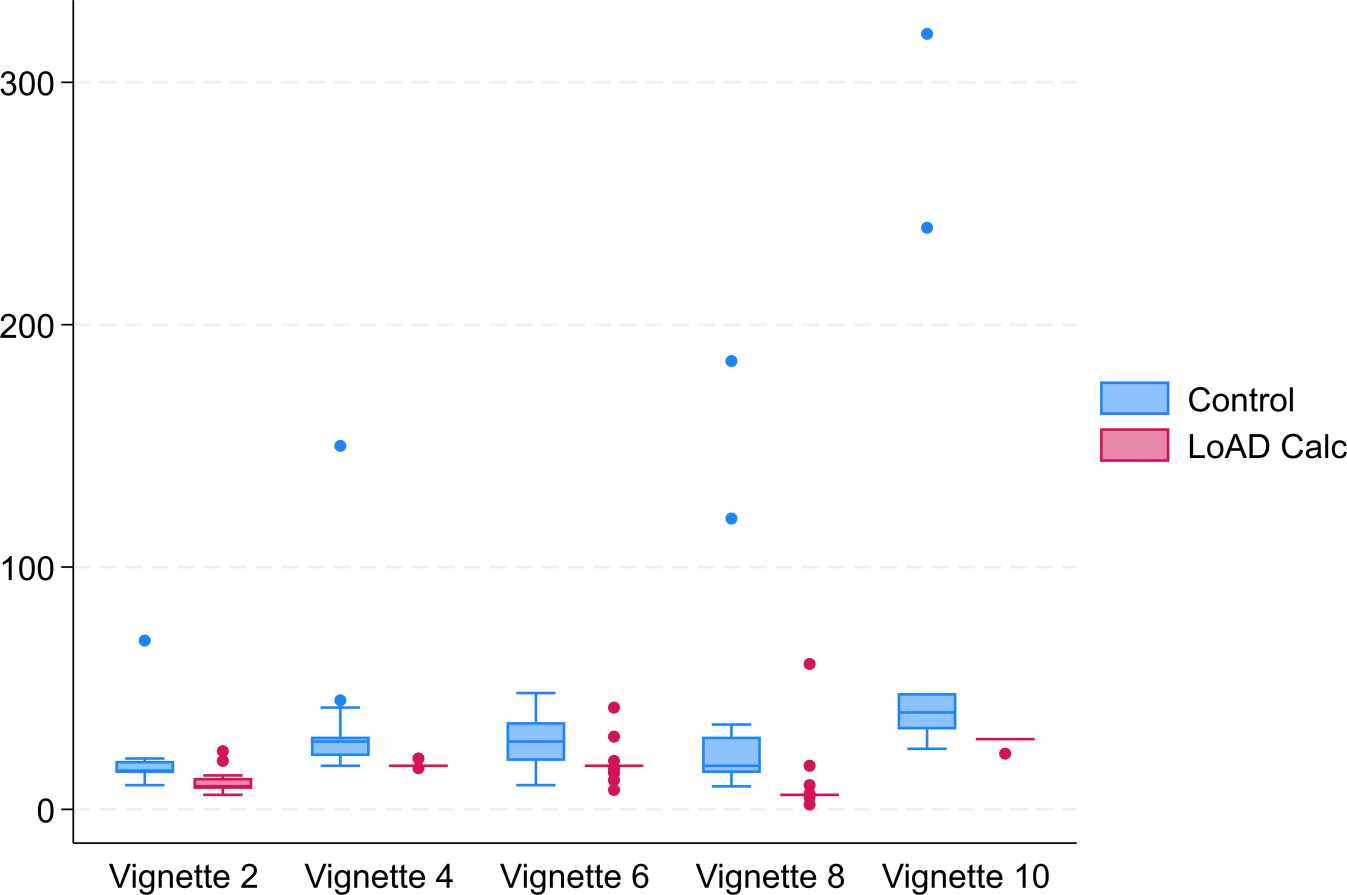
Box plot of the volumes (in mL) recorded by study participants. LoAD Calc: Local Anesthetics Dose Calculator.

Figure 2 (quantities) and Figure 3 (volumes) show that variability was more limited when the LoAD Calc app was used.

## Discussion

### Principal Results

Using the LoAD Calc app significantly reduced overdose rates compared to traditional methods. This difference was consistent across all 3 LAs studied and remained significant even when using simplified calculation methods based solely on AW or IBW. These results demonstrate that the app’s benefit is not an artifact of the chosen reference standard but extends to more commonly used calculation approaches, confirming the initial hypothesis that this mHealth app supports the safer calculation of maximum LA doses in simulated scenarios without increasing the risk of underdosing.

The reduction in overdose rates observed using LoAD Calc could contribute to reducing the risk of LAST, even though potentially unsafe doses do not systematically result in toxicity [19]. By standardizing dose calculations and systematically integrating patient-specific risk factors, the app could constitute an additional safety barrier in locoregional anesthesia practice. In addition, this mHealth app could also reduce interpractitioner variability and contribute to improving the overall quality and standardization of care [20].

Several tools have been developed to assist practitioners in calculating LA doses. The Williams nomogram and the WiMP formula (weight×maximum dose/percentage concentration) [9,21], while useful, do not allow for the integration of all patient-specific variables necessary for optimal calculation. Similarly, existing mobile apps such as MDCalc’s Anesthetic Dosing Calculator [22], the Podiatry Institute’s LA Toxic Dose Calculator [23], and SafeLocal by Johns Hopkins Digital calculate doses based on actual body weight without adjustment [24], do not account for comorbidities or drug interactions, and have not been validated for clinical practice. Preliminary assessments of these apps suggest that most permit the calculation of doses exceeding safe limits and could potentially lead to LAST [10].

Regarding weight calculation recommendations, there is currently significant heterogeneity in the literature. Different weight calculation methods have been proposed, including IBW calculated using formulas such as Devine [9], adjusted body weight [25], or lean body weight as recommended by the 3rd ASRA Practice Advisory [6], which can be calculated using the Boer formula [26]. These variations highlight the need for clarification and standardization of recommendations. The rules integrated into LoAD Calc, although very restrictive and prioritizing safety, may require adaptation based on the evolution of international consensus to promote wider adoption.

In contrast to these existing tools, the LoAD Calc app distinguishes itself by its ability to simultaneously integrate multiple patient-specific parameters: IBW, cardiac, renal or hepatic insufficiency, pregnancy, advanced age, and potential drug interactions. Each parameter is clearly defined in the app, enabling standardized use. Moreover, unlike nomograms and other apps that do not address this complexity, LoAD Calc allows the calculation of maximum safe doses for a mixture of 2 LAs, a particularly useful functionality in clinical practice but complex to perform mentally [27].

The results also demonstrate excellent app usability, with a median SUS score of 98 (IQR 95-100), reflecting very high user satisfaction. This high usability likely stems from the iterative user-centered design approach employed during app development [28], which prioritized clinicians’ needs and workflow integration. App use did not increase the time required to complete calculations, a crucial element for clinical acceptability [29]. Furthermore, participants reported significantly higher confidence in their calculations when using the app, suggesting that the tool may enhance clinicians’ situational awareness and help avoid calculation errors during locoregional anesthesia procedures.

The mHealth apps have become an integral part of modern clinical practice and are already well adopted by anesthesiologists [30]. These tools enable rapid access to information, particularly at the patient’s bedside, and offer valuable support for improving daily medical practice and decision-making [31]. By standardizing complex calculations and reducing cognitive load, such apps may also decrease practitioner stress [32], which could ultimately improve both the quality of care and clinician well-being.

Several perspectives for future development, research, and implementation can be envisioned. The LoAD Calc app is currently based on 3 LAs, namely lidocaine, levobupivacaine, and ropivacaine, and could be expanded to include additional agents. Future versions could also allow mixing of more than 2 LAs and incorporate repeated doses with time intervals, common scenarios in obstetric anesthesia or when combining spinal anesthesia with peripheral nerve blocks. Integration with electronic medical records could facilitate the automatic retrieval of patient data. While currently targeting anesthesiologists, the tool could benefit other specialties using LAs daily, such as surgeons, ophthalmologists, or dentists, though adaptation to their specific practices would be necessary. Real-world observational studies are necessary to evaluate the app’s impact on clinical outcomes, particularly LAST incidence, as well as user satisfaction and sense of safety. Long-term evaluation should assess adoption patterns, adherence factors, and the standardization of dose calculations in practice. Multicenter studies would establish the generalizability of results across different contexts, while cost-effectiveness analysis would guide larger-scale implementation decisions. For wider adoption beyond research settings, the app would require recognition as a medical device with appropriate regulatory approval.

### Limitations

Several limitations must be acknowledged. First, the trial was conducted exclusively within the Anesthesiology Department of Geneva University Hospitals over a limited period. Since all participating physicians belonged to the same institution, their responses may have been influenced by shared departmental practices or organizational culture, which may limit the generalizability of the results. Second, participants were placed in a simulated environment using clinical vignettes. Therefore, real-world clinical conditions might influence how clinicians calculate maximum LA doses differently than observed in this controlled setting. Time pressure, frequent interruptions, and the complexity of real clinical situations could modify observed performances. Third, some observed outliers with unexpectedly high values likely resulted from participants confusing milligram and milliliter units when entering their answers, despite clear instructions specifying which unit to use for each vignette. A post hoc sensitivity analysis excluding these participants confirmed that our primary conclusions were not affected by these extreme values. Another limitation lies in the choice to include only anesthesiologists, either in training or already certified, many of whom use LAs daily. The perception and use of the LoAD Calc app could differ in other medical specialties with different practices and needs regarding local anesthesia. Extending app use will therefore require understanding the specific needs of other clinical disciplines and adapting the tool accordingly.

### Conclusion

This study demonstrates that LoAD Calc, an mHealth app designed to calculate maximum safe LA doses including mixtures, significantly reduces calculation-related overdose rates in simulated scenarios compared to traditional calculation methods, without increasing calculation time and with excellent usability. Clinicians also reported greater confidence when using LoAD Calc compared to traditional methods. These findings suggest that the app may support safer and more standardized LA dose calculations in clinical practice. However, as this was a vignette-based simulation study, further research in real-world clinical settings is needed to determine whether these improvements translate into enhanced patient safety and reduced risk of LAST.

## Supplementary material

10.2196/89236 Multimedia Appendix 1 Screenshots of the LoAD Calc app.

10.2196/89236 Multimedia Appendix 2 Clinical vignettes.

10.2196/89236 Checklist 1 CONSORT-eHEALTH checklist (V 1.6).

## Abbreviations

ASRA: American Society of Regional Anesthesia and Pain Medicine
AW: actual weight
CCER: Commission Cantonale d'Éthique de la Recherche
CHERRIES: Checklist for Reporting Results of Internet E-Surveys
CONSORT-eHEALTH: Consolidated Standards of Reporting Trials Consolidated Standards of Reporting Trials of Electronic and Mobile Health Applications and Online Telehealth
IBW: ideal body weight
LA: local anesthetic
LAST: local anesthetic systemic toxicity
LoAD Calc: Local Anesthetics Dose Calculator
RR: relative risk
