# Supplementary figures and images for: Evaluation of the Impact of a Mobile App (LoAD Calc) on the Calculation of Maximum Safe Doses of Local Anesthetics: Randomized Controlled Trial
Source: JMIR Mhealth Uhealth. 2026 Jul 30;14:e89236. doi: 10.2196/89236 (PMC13422587; doi:10.2196/89236)

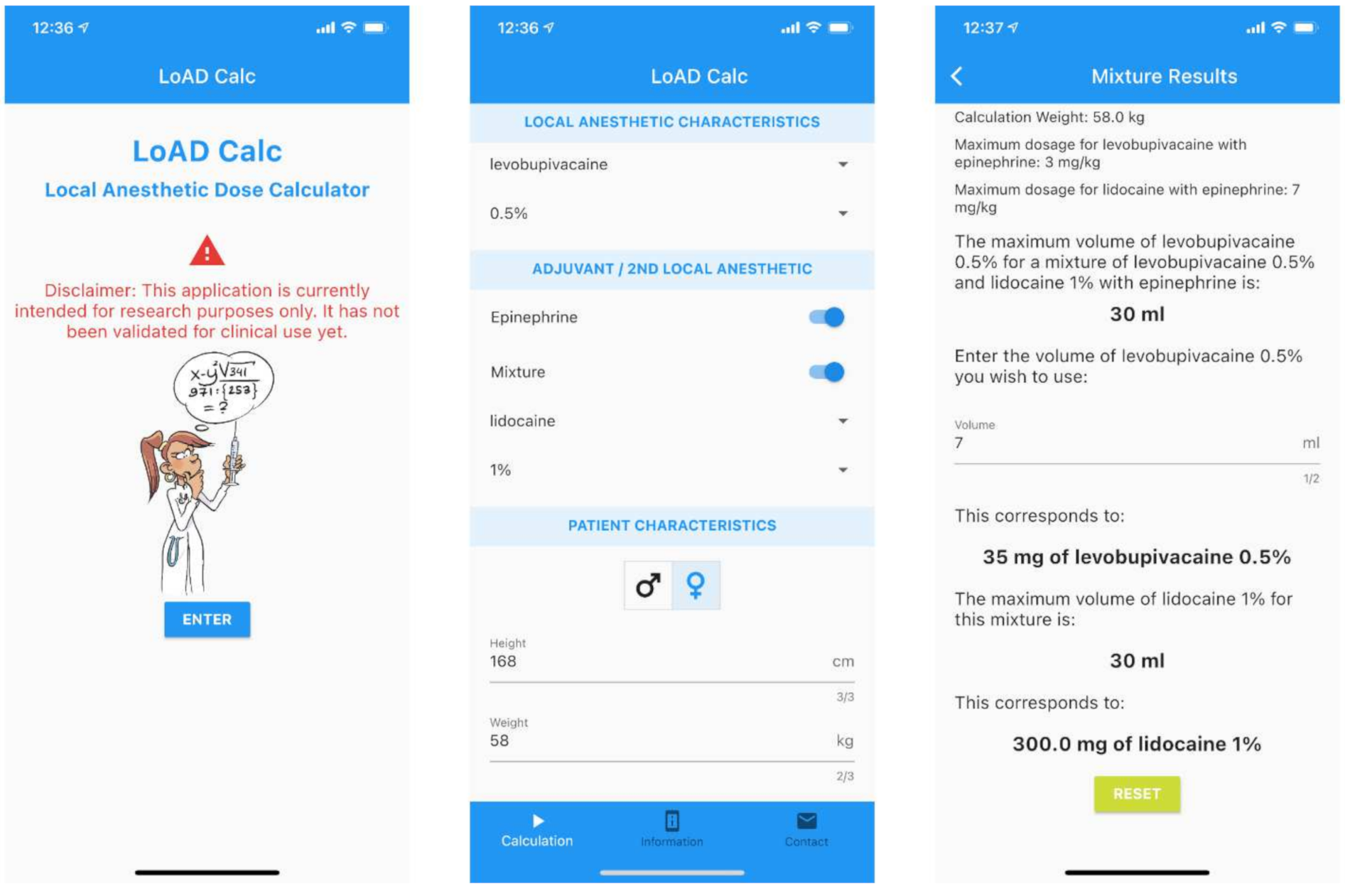

Supplement: Multimedia Appendix 1 [file mhealth-v14-e89236-s001.png]
