# Supplementary material for: Evaluation of the Impact of a Mobile App (LoAD Calc) on the Calculation of Maximum Safe Doses of Local Anesthetics: Randomized Controlled Trial
Source: JMIR Mhealth Uhealth. 2026 Jul 30;14:e89236. doi: 10.2196/89236 (PMC13422587; doi:10.2196/89236)
Supplement: Multimedia Appendix 2 [file mhealth-v14-e89236-s002.docx]

| ***Original (French) version*** | ***English translation*** |
| --- | --- |
| L’exercice consiste à **déterminer la dose maximale** que vous pourriez donner à des patient-e-x-s spécifiques. Les **vignettes sont donc des prétextes** à l’utilisation d’anesthésiques locaux dans divers contextes et il ne s’agit pas de débattre sur le choix de l’anesthésique local ou de la technique anesthésique. | The goal is to **determine the maximum dose** you could give to specific patients. The **vignettes are therefore pretexts for** the use of local anesthetics in a variety of contexts. You are not expected to debate the choice of local anesthetic or anesthetic technique. |

## Cas N°1 / Case N°1

| ***Original (French) version*** | ***English translation*** |
| --- | --- |
| Homme de 33 ans (55kg pour 174cm), ASA 2, connu pour tabagisme actif 15 UPA et dénutrition, autrement en bonne santé habituelle. La fonction hépatique et la fonction rénale sont normales. Il n’a pas de comorbidités cardiaques ni respiratoires. Le patient n’a pas de traitement habituel. Il est prévu pour une chirurgie ligamentaire de l’épaule gauche.  Vous optez, en accord avec le patient, pour un bloc interscalénique suivi d’une anesthésie générale.  Quelle est la dose maximale (**en milligrammes**) de ropivacaïne 0.5% que vous pourriez injecter pour effectuer ce bloc chez ce patient ? | 33-year-old man (55kg for 174cm), ASA 2, active smoker (15 UPA), in usual good health except undernutrition. Liver and renal function are normal. He has no cardiac or respiratory comorbidities. The patient does not take any medication. He is scheduled for ligament surgery of the left shoulder.  In agreement with the patient, you opt for an interscalene block followed by general anesthesia.  What is the maximum dose (**in milligrams)** of ropivacaine 0.5% that you could inject to perform this block on this patient? |

## Cas N°2 / Case N°2

| ***Original (French) version*** | ***English translation*** |
| --- | --- |
| Femme de 74 ans (51.5kg pour 159cm), ASA 2, connue pour hypertension artérielle, reflux gastro-œsophagien et hypercholestérolémie. La fonction hépatique et la fonction rénale sont normales. Elle n’a pas d’autres comorbidités cardiaques ou respiratoires. Son traitement habituel se compose de Co-Lisinopril (lisinopril et hydrochlorothiazide) et d’atorvastatine. Elle est prévue pour une prothèse totale du genou gauche.  La stratégie anesthésique retenue, en accord avec la patiente, est un bloc fémoral (avec de la ropivacaïne 0.5%) ainsi qu’un bloc du plexus poplité (avec de la lévobupivacaïne 0.5%) avant une anesthésie générale.  Vous avez déjà effectué le bloc fémoral en utilisant 10 ml de ropivacaïne 0.5%. Quel est le volume maximal (**en millilitres**) de lévobupivacaïne 0.5% que vous pourriez injecter pour effectuer le bloc du plexus poplité chez cette patiente ? | 74-year-old woman (51.5kg for 159cm), ASA 2, known for hypertension, gastroesophageal reflux and hypercholesterolemia. Liver and renal function are normal. She has no other cardiac or respiratory comorbidities. Her usual treatment consists of Co-Lisinopril (lisinopril and hydrochlorothiazide) and atorvastatin. She is scheduled for a total left knee replacement.  The anesthetic strategy chosen, in agreement with the patient, was a femoral block (with ropivacaine 0.5%) and a popliteal plexus block (with levobupivacaine 0.5%) prior to general anesthesia.  You have already performed the femoral block using 10 ml ropivacaine 0.5%. What is the maximum volume (**in milliliters**) of levobupivacaine 0.5% that you could inject to perform the popliteal plexus block in this patient? |

## Cas N°3 / Case N°3

| ***Original (French) version*** | ***English translation*** |
| --- | --- |
| Femme de 32 ans (60kg pour 170cm), ASA 2 pour première grossesse harmonieuse actuellement à 30 semaines d’aménorrhée. La fonction hépatique et la fonction rénale sont normales. Elle n’a pas de comorbidités cardiaques ni respiratoires. La patiente n’a pas de traitement habituel. Elle est prévue pour une ostéosynthèse du poignet gauche suite à une fracture de l’extrémité distale du radius. Vous optez, au vu du contexte clinique et en accord avec la patiente, pour un bloc axillaire.  L’infirmière a déjà ouvert 2 ampoules de 10ml de ropivacaïne 0.5% que vous décidez d’utiliser entièrement. Vous préférez toutefois ajouter de la lidocaïne 1% afin de pouvoir vérifier rapidement l’efficacité du bloc. Quelle est la dose maximale (**en milligrammes**) de lidocaïne 1% que vous pourriez injecter (en plus des 20ml de ropivacaïne 0.5%) pour effectuer ce bloc chez cette patiente ? | 32-year-old woman (60kg for 170cm), ASA 2 for first harmonious pregnancy currently at 30 weeks' amenorrhea. Liver and renal function are normal. She has no cardiac or respiratory comorbidities. She doesn’t take any medication. She is scheduled for osteosynthesis of the left wrist following a fracture of the distal end of the radius. Taking the clinical context into account and in agreement with the patient, you opt for an axillary block.  The nurse has already opened 2 vials of 10ml ropivacaine 0.5% which you decide to use entirely. You would aldo like to add lidocaine 1% so that you can quickly check the effectiveness of the block. What is the maximum dose (**in milligrams**) of lidocaine 1% that you could inject (in addition to the 20ml of ropivacaine 0.5%) to perform this block on this patient? |

## Cas N°4 / Case N°4

| ***Original (French) version*** | ***English translation*** |
| --- | --- |
| Homme de 75 ans (70kg pour 170cm), ASA 2, connu pour hypertension artérielle et insuffisance rénale chronique avec un débit de filtration glomérulaire estimé (eGFR) à 30 ml/min. La fonction hépatique est normale. Il n’a pas de comorbidités cardiaques ni respiratoires. Dans son traitement habituel il prend du lisinopril, de l’amlodipine et du torasémide. Il est prévu pour une arthroscopie du genou gauche.  Comme stratégie anesthésique vous optez, en accord avec le patient, pour un bloc saphène (avec de la lévobupivacaïne 0.5%) avant une anesthésie générale.  Quel est le volume maximal (**en millilitres**) de lévobupivacaïne 0.5% que vous pourriez injecter pour effectuer ce bloc chez ce patient ? | 75-year-old man (70kg for 170cm), ASA 2, known for hypertension and chronic renal failure with an estimated glomerular filtration rate (eGFR) of 30 ml/min. His liver function tests are normal. He has no cardiac or respiratory comorbidities. His usual treatment includes lisinopril, amlodipine and torasemide. He is scheduled for arthroscopy of the left knee.  In agreement with the patient, you opt for a saphenous vein block (with 0.5% levobupivacaine) prior to general anesthesia.  What is the maximum volume (**in milliliters)** of levobupivacaine 0.5% that you could inject to perform this block on this patient? |

## Cas N°5 / Case N°5

| ***Original (French) version*** | ***English translation*** |
| --- | --- |
| Femme de 37 ans (120kg pour 180cm), ASA 2, connue pour hypertension artérielle et obésité. La fonction hépatique et la fonction rénale sont normales. Elle n’a pas d’autres comorbidités cardiaques ou respiratoires. Elle est au décours d’une infection basse des voies urinaires traitée par ciprofloxacine (dernière prise prévue ce jour). Elle ne prend que de l’enalapril 10mg 1x/j comme traitement habituel. Elle est prévue pour une stabilisation d’une fracture de la cheville.  Vous décidez d’effectuer, en accord avec la patiente, un bloc sciatique poplité (lévobupivacaïne 0.5%) avant une anesthésie générale.  Quelle est la dose maximale (**en milligrammes**) de lévobupivacaïne 0.5% que vous pourriez injecter pour effectuer ce bloc chez cette patiente ? | 37-year-old woman (120kg for 180cm), ASA 2, known for hypertension and obesity. Liver and renal function are normal. She has no other cardiac or respiratory comorbidities. She is recovering from a lower urinary tract infection treated with ciprofloxacin (with the last scheduled dose today). She only takes enalapril 10mg once daily. She is scheduled for stabilization of an ankle fracture.  In agreement with the patient, you decide to perform a popliteal sciatic block (levobupivacaine 0.5%) prior to general anesthesia.  What is the maximum dose (**in milligrams)** of levobupivacaine 0.5% that you could inject to perform this block on this patient? |

## Cas N°6 / Case N°6

| ***Original (French) version*** | ***English translation*** |
| --- | --- |
| Femme de 65 ans (80kg pour 175cm), ASA 3, connue pour bronchopneumopathie chronique obstructive GOLD 2 et cirrhose hépatique Child B sur stéatose hépatique non-alcoolique (bilirubine 40 µmol/l, albumine 30 g/l, TP 45%, thrombocytes 150 G/l, pas d’ascite ni d’encéphalopathie), hypercholestérolémie et diabète de type 2 non insulino-requérant. Une consultation d’hémostase a été effectuée et ne relève pas de contre-indication à une anesthésie loco-régionale, la perturbation du TP étant due à la progression de la maladie hépatique uniquement. La fonction rénale est normale. La patiente n’a pas de comorbidités cardiaques. Dans son traitement habituel elle prend de l’atorvastatine, de la metformine et du Spiriva. Elle est prévue pour une prothèse totale de genou gauche.  Comme stratégie anesthésique vous optez, en accord avec la patiente, pour un bloc fémoral (avec 12ml de lévobupivacaïne 0.5%) ainsi qu’un bloc du plexus poplité (avec de la ropivacaïne 0.375%) avant une anesthésie générale.  Vous avez déjà effectué le bloc fémoral en utilisant 12 ml de lévobupivacaïne 0,5%. Quel est le volume maximal (**en millilitres**) de ropivacaïne 0.375% que vous pourriez injecter pour effectuer le bloc du plexus poplité chez cette patiente ? | 65-year-old woman (80kg for 175cm), ASA 3, known for GOLD 2 chronic obstructive pulmonary disease and Child B hepatic cirrhosis consecutive to non-alcoholic steatohepatitis (bilirubin 40 µmol/l, albumin 30 g/l, PT 45%, thrombocytes 150 G/l, no ascites or encephalopathy), hypercholesterolemia and non-insulin-requiring type 2 diabetes. A hemostasis consultation found no contraindication to locoregional anesthesia, the disturbance in PT being due solely to the progression of liver disease. Renal function tests are normal. The patient has no cardiac comorbidities. Her usual treatment includes atorvastatin, metformin and Spiriva. She is scheduled for a total left knee replacement.  In agreement with the patient, you opt for a femoral block (with 12ml of levobupivacaine 0.5%) and a popliteal plexus block (with ropivacaine 0.375%) prior to general anesthesia.  You have already performed the femoral block using 12 ml of levobupivacaine 0.5%. What is the maximum volume (**in milliliters**) of ropivacaine 0.375% that you could inject to perform the popliteal plexus block in this patient? |

## Cas N°7 / Case N°7

| ***Original (French) version*** | ***English translation*** |
| --- | --- |
| Homme de 80 ans (60kg pour 175cm), ASA 3, connu pour fibrillation auriculaire paroxystique, s/p TAVI (Transcatheter Aortic Valve Implantation) il y a 1 an pour une sténose aortique sévère avec fraction d’éjection ventriculaire gauche à 30%, s/p accident vasculaire cérébrale sylvien gauche non séquellaire, hypertension et hypercholestérolémie. Il est actif et indépendant pour les activités de la vie quotidienne. La fonction hépatique et la fonction rénale sont normales. Dans son traitement habituel il prend du Co-Lisinopril (lisinopril et hydrochlorothiazide), de l’atorvastatine et du rivaroxaban actuellement en suspens. Il est prévu pour une prothèse céphalique de la hanche gauche.  Vous optez, en accord avec le patient (qui refuse catégoriquement une anesthésie neuraxiale), pour un bloc fémoral et du nerf cutané latéral de la cuisse (avec de la lévobupivacaïne 0.375%) avant une anesthésie générale.  Quelle est la dose maximale (**en milligrammes**) de lévobupivacaïne 0.375% que vous pourriez injecter pour effectuer ce bloc chez ce patient ? | 80-year-old man (60kg for 175cm), ASA 3, known for paroxysmal atrial fibrillation, who underwent TAVI (Transcatheter Aortic Valve Implantation) 1 year ago for severe aortic stenosis with left ventricular ejection fraction at 30%. He is also known for a prior left sylvian stroke (no sequelae), hypertension and hypercholesterolemia. He is active and independent for activities of daily living. Liver and renal function are normal. His usual treatment includes Co-Lisinopril (lisinopril and hydrochlorothiazide), atorvastatin and rivaroxaban, with the latter currently on hold. He is scheduled for a cephalic left hip replacement.  In agreement with the patient (who categorically refuses neuraxial anesthesia), you opt for a femoral and lateral thigh cutaneous nerve block (with levobupivacaine 0.375%) prior to general anesthesia.  What is the maximum dose (**in milligrams)** of levobupivacaine 0.375% that you could inject to perform this block on this patient? |

## Cas N°8 / Case N°8

| ***Original (French) version*** | ***English translation*** |
| --- | --- |
| Femme de 57 ans (75kg pour 170cm), ASA 1, en bonne santé habituelle. La fonction hépatique et la fonction rénale sont normales. Elle n’a pas de comorbidités cardiaques ni respiratoires. Elle ne prend pas de traitement habituel. Elle est prévue pour une ostéosynthèse de la cheville droite. Comme stratégie anesthésique vous optez, en accord avec la patiente, pour un bloc sciatique poplité (avec de la lévobupivacaïne 0.5%) et un bloc saphène (avec de la lidocaïne 1%).  Vous avez déjà effectué le bloc sciatique poplité aven injectant 15ml de lévobupivacaïne. Quel est le volume maximal (**en millilitres**) de lidocaïne 1% que vous pourriez injecter pour effectuer le bloc saphène chez cette patiente ? | 57-year-old woman (75kg for 170cm), ASA 1, in usual good health. Liver and renal function are normal. She has no cardiac or respiratory comorbidities. She does not take any medication. She is scheduled for osteosynthesis of the right ankle. In agreement with the patient, you opt for a popliteal sciatic block (with levobupivacaine 0.5%) and a saphenous block (with lidocaine 1%).  You have already performed the popliteal sciatic block by injecting 15ml of levobupivacaine. What is the maximum volume (**in milliliters**) of 1% lidocaine you could inject to perform the saphenous vein block on this patient? |

## Cas N°9 / Case N°9

| ***Original (French) version*** | ***English translation*** |
| --- | --- |
| Femme de 84 ans (90kg pour 160cm), ASA 2, connue pour hypertension artérielle et obésité. Son hypertension est actuellement non traitée en raison d’épisodes hypotensifs avec chutes sous le précédent anti-hypertenseur. La fonction hépatique et la fonction rénale sont normales. Elle n’a pas d’autres comorbidités cardiaques ou respiratoires. Elle est prévue pour une prothèse d’épaule droite.  Comme stratégie anesthésique vous optez, en accord avec la patiente, pour un bloc interscalénique (avec de la ropivacaïne 0.375%) suivi d’une anesthésie générale.  Quelle est la dose maximale (**en milligrammes**) de ropivacaïne 0.375% que vous pourriez injecter pour effectuer ce bloc chez cette patiente ? | 84-year-old woman (90kg for 160cm), ASA 2, known for hypertension and obesity. Her hypertension is currently untreated due to hypotensive episodes with falls under the previous anti-hypertensive. Liver and renal function are normal. She has no other cardiac or respiratory comorbidities. She is scheduled for a right shoulder prosthesis.  In agreement with the patient, you opt for an interscalene block (with ropivacaine 0.375%) followed by general anesthesia.  What is the maximum dose (**in milligrams)** of ropivacaine 0.375% that you could inject to perform this block on this patient? |

## Cas N°10 / Case N°10

| ***Original (French) version*** | ***English translation*** |
| --- | --- |
| Femme de 35 ans (80kg pour 157cm), ASA 2, connue pour un diabète de type 1 sous pompe d'insuline. Elle n'a pas de comorbidités cardiaques ou respiratoires. Les fonctions hépatique et rénale sont normales. Elle est prévue pour une chirurgie du pied. En accord avec elle vous optez pour un bloc de cheville (avec de la ropivacaïne 0.5%).  Quel est le volume maximal (**en millilitres**) de ropivacaïne 0.5% que vous pourriez injecter pour effectuer ce bloc chez cette patiente ? | 35-year-old woman (80kg for 157cm), ASA 2, known to have type 1 diabetes on insulin pump. She has no cardiac or respiratory comorbidities. Liver and renal function are normal. She is scheduled for foot surgery. In agreement with her, you opt for an ankle block (with ropivacaine 0.5%).  What is the maximum volume (**in milliliters)** of ropivacaine 0.5% that you could inject to perform this block on this patient? |
